# The molecular neurobiology and neuropathology of opioid use disorder

**DOI:** 10.1016/j.crneur.2021.100023

**Published:** 2021-10-14

**Authors:** Christopher A. Blackwood, Jean Lud Cadet

**Affiliations:** Molecular Neuropsychiatry Research Branch, NIH/NIDA Intramural Research Program, 251 Bayview Boulevard, Baltimore, MD, 21224, USA

**Keywords:** Heroin, Oxycodone, Fentanyl, Methadone, Neuroimaging, Postmortem

## Abstract

The number of people diagnosed with opioid use disorder has skyrocketed as a consequence of the opioid epidemic and the increased prescribing of opioid drugs for chronic pain relief. Opioid use disorder is characterized by loss of control of drug taking, continued drug use in the presence of adverse consequences, and repeated relapses to drug taking even after long periods of abstinence. Patients who suffer from opioid use disorder often present with cognitive deficits that are potentially secondary to structural brain abnormalities that vary according to the chemical composition of the abused opioid. This review details the neurobiological effects of oxycodone, morphine, heroin, methadone, and fentanyl on brain neurocircuitries by presenting the acute and chronic effects of these drugs on the human brain. In addition, we review results of neuroimaging in opioid use disorder patients and/or histological studies from brains of patients who had expired after acute intoxication following long-term use of these drugs. Moreover, we include relevant discussions of the neurobiological mechanisms involved in promoting abnormalities in the brains of opioid-exposed patients. Finally, we discuss how novel strategies could be used to provide pharmacological treatment against opioid use disorder.

## Highlights

• Brain abnormalities caused by opioid intoxication.
• Intoxication of opioids leads to defects in brain neurocircuitries.
• Insight into the molecular mechanisms associated with craving in heroin addicts.

## Graphical abstract

**Figure:**
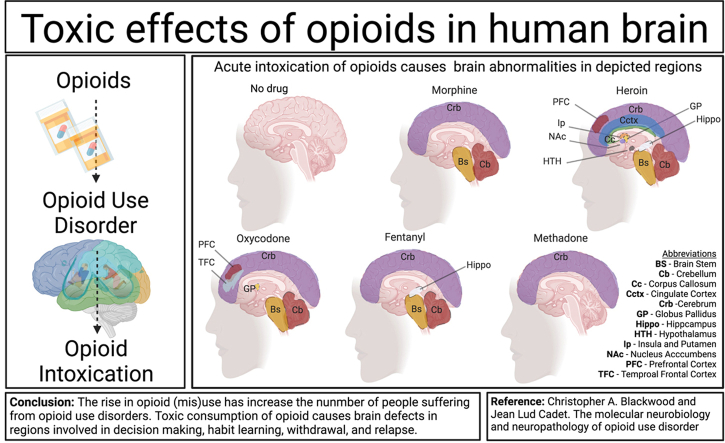

## 1 Introduction

The prolific (mis)use of opioids in the United States (U.S.) and many other countries has created a large number of individuals suffering from opioid use disorder (OUD). OUD is a chronic relapsing disorder driven by neurocircuits that modulate adverse emotional states and trigger relapses (Strang et al., 2020). OUD is also characterized by compulsive drug taking, craving for opioids, and continued opioid intake despite adverse life consequences (Table S1). Although much of the initial study of the neurobiology of drug addiction focused on the acute impact of opioids on the brain using preclinical models, key issues that still need more attention are the long-lasting and potentially toxic effects of opioids on brain structures and their structural consequences on the neurocircuitry that drives the vicious cycle of addiction in people suffering from OUD.

The cost of OUD has led to global health and financial crises, fueled by over-prescription of opioids, increases in recreational opioid use, and expansion of opium cultivation and supplies in various world markets (Banta-Green et al., 2009; UNODC, 2019). In 2018, the U.S. reported that 3.7% of the population over the age of 12 had experimented with opioids (SAMHSA, 2019). That same year, the U.S. reported 46,802 opioid-related fatalities (Hedegaard, 2020). Furthermore, the projected cost of OUD-related problems is approximately $78.5 billion, a number that includes expenditures in healthcare, the criminal justice system, and substance abuse treatment programs (Florence et al., 2016).

Physicians commonly prescribe opioids to manage moderate to severe pain. However, some patients consume opioids to experience euphoria, tranquility, and sedation (Schuckit, 2016). The (mis)use of opioids causes adverse effects such as psychological/physical dependency, continuous relapses, and withdrawal symptoms (Schuckit, 2016) and can lead to overdose (Schiller et al., 2021). Furthermore, compulsive drug takers often develop tolerance (Morrone et al., 2017), which is associated with a need to consume greater amount of drugs to experience the desired effects, thus perpetuating the vicious cycle of addiction.

The behavioral effects of opioids are mediated by their binding to opioid receptors that are located in various regions of the peripheral nervous system (PNS) (Liu et al., 2020) and central nervous system (CNS) (Cumming et al., 2019). Mu, delta and kappa opioid receptors are G-protein-coupled receptors (GPCR) expressed throughout the brain (Hirvonen et al., 2009; Naganawa et al., 2015; Weerts et al., 2008) and the periphery (Mansour and Watson, 1993). Upon stimulation by an agonist, these GPCRs form homo- and heterodimeric complexes to activate G proteins and transduce intercellular signals via a wide range of intracellular pathways in the PNS and CNS. These include the mitogen-activated protein kinase phosphorylation (MAPK) cascade (Blackwood et al., 2021; Deng et al., 2019). Opioid receptors can signal through Gαi/o subunits, inhibit activity of adenyl cyclase, and reduce cyclic AMP production. Stimulation of opioid receptors can also results in the activation of potassium ion channels (Christie et al., 1987), which have been shown to have inhibitory effects on neurons (Torrecilla et al., 2002). Opioid receptor stimulation can also regulate calcium activity at various nerve terminals (Rusin et al., 1997).

**Fig. 1:**
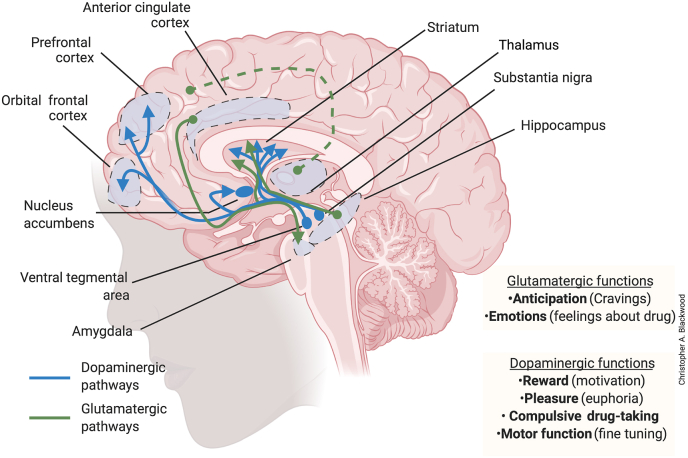
**A model of the brain reward system.** Sagittal illustration of a human brain depicting the dopaminergic pathways (blue) including the ventral tegmental area to the nucleus accumbens, striatum or frontal cortex; and the substantia nigra to the striatum. Glutamatergic pathways (green) are shown from the anterior cingulate cortex to the amygdala and striatum; hippocampus to the striatum; and the cortico-thalamo-cortical interactions. (For interpretation of the references to colour in this figure legend, the reader is referred to the Web version of this article.)

Several synthetic opioids are agonists at the mu opioid receptor that is encoded by the gene OPRM1 (Y. Chen, Mestek, Liu, Hurley and Yu, 1993). Mu opioid receptors are located in various regions of the human body including vascular system, gastrointestinal tract, immune cells, the PNS, and the CNS (Brejchova et al., 2020; Hirvonen et al., 2009; Kuhar et al., 1973; Mosinska et al., 2016). In humans, mu opioid receptors have very high levels of expression in the amygdala, anterior cingulate cortex, cerebellum, nucleus accumbens, hippocampus, thalamus, and amygdala (Hirvonen et al., 2009; Johansson et al., 2019), with the highest densities observed in the striatum, thalamus, and cingulate cortex (Nummenmaa et al., 2018). Mu opioid receptors modulate various behavioral functions including reward, mood, anxiety, neuroendocrine function, and gastrointestinal motility (Nummenmaa and Tuominen, 2018; Pecina et al., 2019). Mu opioid receptors have also been implicated in various aspects of substance use disorders (Blackwood et al., 2019; Blackwood et al., 2019; Gorelick et al., 2008; Meye et al., 2012), in part, via interactions with glutamatergic and dopaminergic pathways (Chartoff and Connery, 2014; Hearing, 2018). Specifically, administration of opioids induces dopamine release in dopaminergic reward-related structures (Di Chiara and Imperato, 1988; Johnson and North, 1992). However, there are reports that dopamine in the thalamus performs a limited role in rodents compared to primate and human brain (Garcia-Cabezas et al., 2009; Garcia-Cabezas et al., 2007). Nevertheless, repeated exposure to drugs may lead to alterations in these structures, with these molecular and cellular neuroadaptations being responsible for the various manifestations of OUD including compulsive drug taking and craving (Fig. 1).

In what follows, we summarize neurobiological and neuropathological changes associated with exposure to several opioid drugs including oxycodone, morphine, heroin, methadone and fentanyl.

## 2 Oxycodone use disorder

In 2018, 9.9 million people aged 12 older were found to (mis)use prescription opioids, including oxycodone (SAMHSA, 2019). In the same year, prescription opioids were responsible for 14,975 overdose deaths (Wilson et al., 2020). Oxycodone is a semisynthetic opioid that is synthesized mainly from thebaine, an alkaloid found in opium poppy (Berenyi et al., 2009). Physicians prescribe oxycodone to treat moderate to severe pain largely from cancer and postsurgical operations. The short-term effects of oxycodone include euphoria and relaxation (Remillard et al., 2019). Long-term use can result in confusion, drowsiness, and respiratory complications (Fox et al., 2018). The repeated use of oxycodone increases tolerance and is associated with severe withdrawal and repeated relapses even during treatment for addiction (Schuckit, 2016). Oxycodone is structurally similar to morphine, but contains a methyl functional group enabling its faster penetration into the blood brain barrier (Bostrom et al., 2008) Oxycodone is metabolized into nor-oxycodone and oxymorphone (Z. R. Chen, Irvine, Somogyi and Bochner, 1991; Lalovic et al., 2006; Lalovic et al., 2004). In addition to binding of mu opioid receptors, oxycodone and oxymorphone appear to have lower binding affinities for delta opioid receptors (Gendron et al., 2016; Thompson et al., 2004). In addition to its biochemical effects, long-term use is also accompanied with brain abnormalities (Duran et al., 2017; Morales Odia et al., 2010; Odia, Jinka and Ziai, 2010; Wheaton et al., 2019). In what follows, we summarize some of these defects and how they disrupt critical neurocircuits as well as their clinical ramifications.

### 2.1 Biochemical and structural abnormalities

Several investigators have described lesions of the brain caused by toxic levels of oxycodone in children and adults (Duran et al., 2017; Morales Odia et al., 2010; Odia et al., 2010; Wheaton et al., 2019). Magnetic resonance imaging (MRI) of a 4-year old and a 46-year old brains of patients with opioid intoxication revealed leukoencephalopathy in cerebellum (Odia, 2010; Wheaton et al., 2019). Several clinical findings showed that toxic levels of oxycodone caused structural abnormalities in the cerebellum (Duran et al., 2017; Morales Odia et al., 2010; Odia et al., 2010; Wheaton et al., 2019) and in the globus pallidus (Odia, 2010). Radiological findings in postmortem brains from oxycodone addicts showed edema (Wheaton et al., 2019), hypoattenuation (Duran et al., 2017), and hydrocephalus (Wheaton et al., 2019) in the cerebellar regions. Postmortem brains from oxycodone addicts with a history of polydrug use with stimulants showed gray matter and white matter defects in the frontal and temporal cortices as well as neuronal axon damage (Alturkustani et al., 2017).

### 2.2 Oxycodone effects on neurocircuits

Structural abnormalities in the brain may in part contribute to defects in the neural activity found in oxycodone abusers. Brain electrical recording studies in oxycodone abusers have shown altered neural activity in the cortex and basal ganglia (Hansen et al., 2018; Lelic et al., 2016; Lelic et al., 2017). Electroencephalography from patients administered oxycodone showed a decrease in neural activity in the insula and frontal gyrus of the cortex (Lelic et al., 2017). Neural circuits connected to the cingulate cortex showed altered electrical stimulation after exposure to oxycodone (Lelic et al., 2016). Oxycodone users showed decreased functional connectivity between the frontal cortex and structures of the basal ganglia region (Hansen et al., 2018). Decreased neural activity may impact neurocircuits between the prefrontal cortex and nucleus accumbens or within the basal ganglia regions (Fig. S1) that are associated with the process of decision-making and habit learning/reward as illustrated in Fig. S2. These changes in neurocircuits are postulated to cause the progression of chronic relapses.

## 3 Morphine use disorder

Morphine is an organic alkaloid substance produced from opium poppy plants. According to the *World Drug Report* during 2014–2018, more than 89% of opium production is largely concentrated in Afghanistan, Myanmar and Mexico. Moreover, the recent expansion of opium cultivation in Mexico is partially responsible for the increase of illicit use in the U.S. (UNODC, 2019). Unauthorized use of morphine along with improper injection hygiene has largely contributed to high rates of hepatitis, human immunodeficiency viruses and other blood borne illnesses. Morphine has been used in clinical settings to treat pain for several decades. The adverse effects of morphine include respiratory depression and physical or psychological dependence (Cooper et al., 2017). The affects of morphine are based on its ability to be metabolized. Morphine is metabolized into morphine-6-glucuronide and morphine-3-glucuronide (Yoshimura et al., 1973). Morphine-6-glucuronide penetrates the blood-barrier and has a high affinity to mu opioid receptors (De Gregori et al., 2012). Moreover, morphine-6-glucuronide is more potent than morphine-3-glucuronide in its analgesic effects (Frances et al., 1990; Frances et al., 1992) on the CNS.

### 3.1 Biochemical and structural abnormalities

**Fig. 2:**
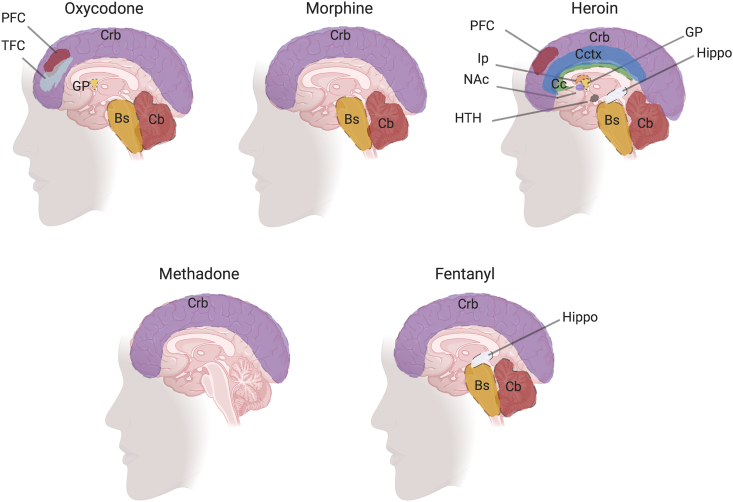
**Brain deficits in human opioid use disorders.** Cartoon illustration of human brain showing areas affected by acute or chronic intoxication of heroin, morphine, oxycodone, methadone and fentanyl. Abbreviations: Bs, Brain stem; Cb, Cerebellum; Crb, Cerebrum; Cctx, Cingulate cortex; Cc, Corpus Callosum; GP, Globus Pallidus; Hippo, Hippocampus; HTH, Hypothalamus; Ip, Insula and putamen; NAc, Nucleus Accumbens; PFC, Prefrontal Cortex.

Clinical reports have shown that morphine intoxication leads to brain defects in corpus callosum, globus pallidus and cerebrum (Duran et al., 2017; Nanan et al., 2000). T1-and T2-weighted images from postmortem brain found enlarged corpus callosum and atrophied cerebellum and cerebrum tissues (Duran et al., 2017; Nanan et al., 2000). Postmortem tissue from morphine users also showed lesions in the spinal root ganglia, which contains the respiratory neurons that play a role in breathing (Martin et al., 1987). Clinical investigations found that morphine users showed hypoxic leukoencephalopathy 2–40 days after an overdose (Nanan et al., 2000; Reisner et al., 2015; Salazar and Dubow, 2012). Computed tomography scans from morphine users showed hypodensities in globus pallidus (Salazar and Dubow, 2012). Other brain defects in morphine users include cerebellar edema, hydrocephalus and cerebellar hypoattenuation (Duran et al., 2017; Reisner et al., 2015) (Fig. 2). Morphine intoxication in infants showed decreased brain volume and cerebral depression (Norman et al., 2013; Tataranno et al., 2020).

### 3.2 Morphine effects on neurocircuits

Administration of morphine in volunteers showed increased neural activity in the frontal, anterior cingulate and insula cortices (Jones et al., 1991). In resting-state functional MRI of patients who were given morphine, were found to have altered brain connectivity in the posterior cingulate and frontal cortices, basal ganglia, and cerebellum (Khalili-Mahani et al., 2012; Kleinloog et al., 2015). Moreover, patients taking morphine showed deficits in brain connectivity that caused defects in perception that were associated with hallucinations (Kleinloog et al., 2015).

## 4 Heroin use disorder

Heroin is a highly addictive natural substance derived from the seedpod of various opium plants largely cultivated in Asia, Mexico and Colombia. In 2018, over 800,000 people that consumed heroin were above 12 years of age (SAMHSA, 2019). During 1999–2018, heroin caused approximately 115,000 overdose deaths (Wilson et al., 2020). 24–36% of all heroin addicts experienced the criminal justice system because of illegal drug use and criminal activities to obtain the drug (Boutwell et al., 2007). Because heroin is an illegal substance many users often reuse needles, which increases the incidence of blood-borne infections, including hepatitis and human immunodeficiency viruses from needle sharing (Reardon, 2019). Heroin mixed with a cutting reagent is also consumed through snorting or smoking.

The acetyl functional groups allow heroin to rapidly pass through the blood brain barrier. In the brain, heroin is metabolized into 6-monacetylmorphine, which has a high affinity for mu opioid receptors. The psychopharmacological effects of heroin include euphoria and the sense of relaxation as well as sleep (NIDA, 2019). Short-term effects are clouded mental functioning and back-and-forth state of being conscious (Louria et al., 1967). Long-term effects are depression and antisocial personality disorder (Louria et al., 1967). Other effects of heroin are tolerance and withdrawal symptoms that occur after long period of abstinence. Furthermore, heroin use causes respiratory depression, which can lead to permanent neurological damage and death (Jolley et al., 2015; Ngai, 1961).

### 4.1 Biochemical and structural abnormalities

Heroin use is linked to several adverse consequences in the human brain (Buttner et al., 2000; Pandria et al., 2018) (Table 1 and Fig. 2). Studies from postmortem brain tissues revealed decrease in volume of hypothalamus (Muller et al., 2018), nucleus accumbens (Muller et al., 2015; Seifert et al., 2015; Tolomeo et al., 2018) and putamen (Bach et al., 2019) structures. Heroin use causes neuronal depletion (Pearson et al., 1976) and structural defects in the globus pallidus (Muller et al., 2019; Tolomeo et al., 2018; Vila and Chamorro, 1997). Heroin addicts showed reduce gray matter density in the frontal, cingulate and occipital cortical regions. The gray matter defects were visible after three days of abstinence, but disappeared after one month, suggesting that this abnormality is recoverable over time (X. Wang et al., 2012).

**Table 1:** Summary of data from human postmortem brains.

Opioid	Brain region	Brain abnormalities	References
**Oxycodone**	Cb and GP	Leukoencephalopathy, cerebellar and GP lesions.	Odia et al. (2010)
	Cb	Cerebellar edema, cerebellar hypoattenuation	Duran et al. (2017)
	Cerebellum, white matter and Crb	Cerebellar edema, hydrocephalus, cerebellar lesions, white matter hyperintensity, and leukoencephalopathy	Wheaton et al. (2019)
**Morphine**	Neuronal defects	Loss of neurons in the spinal root ganglia.	Martin et al. (1987)
	Cb and white matter	Leukoencephalopathy, white matter hyperintensity	Nanan et al. (2000)
	Crb	Cerebellar edema, hydrocephalus and cerebellar hypoattenuation	Duran et al. (2017)
**Heroin**	GP	Neuronal depletion in the GP	Pearson et al. (1976)
	Cb and Crb	Leukoencephalopathy, Cb white mater defects and crb edema	Wolters et al. (1982)
	Cb	Leukoencephalopathy, Cb edema, gray and white mater cerebral defects	Rizzuto et al. (1997)
	GP, Hippo, Crb	bilateral lesions in GP, defects in hippocampus and Crb	Andersen et al., 1999
	Gray matter	Gray matter defects	Wang et al. (2010)
	Cerebellum and Cc	White matter edema, defects in the corpus callosum, and small ventricles	Krinsky and Reichard, 2012
	Cortex and gray matter	Reduced gray matter density in frontal, cingulate and occipital cortices	Wang et al. (2012)
	NAc	Reduced NAc	Muller et al. (2015)
	NAc	Reduced NAc	Seifert et al. (2015)
	HTH	Lesions in HTH	Montoya-Filardi et al., 2016
	White matter	White matter hyperintensity	Short et al., 2017
	HTH	Reduced HTH	Muller et al. (2018)
	GP and NAc	Reduced NAc and GP	Tolomeo et al. (2018)
	Insula and putamen	Reduced gray matter volume in putamen and smaller insula	Bach et al. (2019)
	GP	Reduced GP	Muller et al. (2019)
**Methadone**	Crb	Crb edema	Palmiere et al. (2011)
	Crb	Crb edema	Winklhofer et al. (2014)
**Fentanyl**	Crb	Leukoencephalopathy	Foy et al. (2011)
	Crb	Crb edema	Helander et al., 2016
	Crb	Crb edema	Guerrieri et al. (2017)
	Crb	Crb edema	Geile et al. (2019)
	Crb	Crb edema	Freni et al. (2019)

Cb, Cerebellum; Cc, Corpus Callosum; Crb, Cerebrum; GP, Globus Pallidus; Hippo, Hippocampus.

HTH; Hypothalamus; NAc, Nucleus Accumbens; and PFC, Prefrontal Cortex.

It has also been shown that heroin users sustained brain lesions from drug toxicity (Chang et al., 1997) or oxygen deprivation (Oehmichen et al., 1996). Drug users that inhaled heroin vapors form lesions in the white matter called spongiform leukoencephalopathy (Chang et al., 1997). Spongiform leukoencephalopathy is often caused by lipophilic toxin contaminants released from the heating of heroin (Kass-Hout et al., 2011; Pirompanich and Chankrachang, 2015; Rizzuto et al., 1997; Wolters et al., 1982). Lesions observed in heroin users are mainly secondary to hypoxic-ischemic damage and secondary encephalopathic pathological changes in the brain. These pathological changes occur as a result of complete or partial reduction of cerebral oxygen supply (Adamides et al., 2009; Fitzgerald et al., 2010). Hypoxic-ischemic encephalopathy contributes to the swelling of the cerebrum causing cerebral edema (Oehmichen et al., 1996; Richter et al., 1973), neuronal damage (Wolters et al., 1982) and neuronal depletion (Bayer et al., 2015).

Immunohistopathological studies revealed that leukoencephalopathy and hypoxic-ischemic encephalopathy activate cell types involved in the immune responses to neuronal damage, further indicative of brain injury (Chistiakov et al., 2017; Oehmichen et al., 1996). Furthermore, brain tissue from heroin users showed an increase in the presence of hippocampal *Glial Fibrillary Acid Protein*, a marker for microglial cells, which are activated in response to inflammation and/or local injury to cells (Oehmichen et al., 1996; Rizzuto et al., 1997). Postmortem hippocampal tissue from heroin addicts show increased *Cluster of Differentiation 68* (CD68)-positive monocytes/macrophages (Chistiakov et al., 2017), supporting the notion that heroin intoxication causes severe brain damage.

### 4.2 Neurobiological mechanisms in heroin addicts

**Fig. 3:**
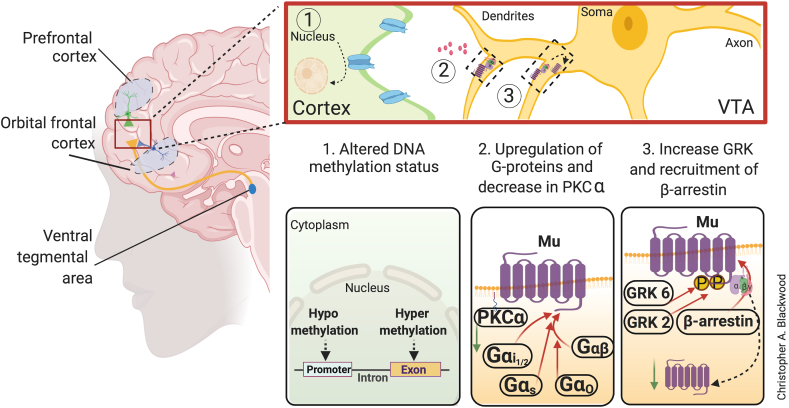
**Potential neurobiological mechanisms associated with craving in heroin addicts.** Decision-making is part of the progression to chronic relapse and is associated with the prefrontal cortex. In postmortem brain heroin causes the (1) altered DNA methylation status in glutamatergic neurons. (2) Increased levels of G-protein subunits: Gαi_1/2_, Gαo, Gαs, and Gαβ. (3) Another possible mechanism involves the upregulation of GRK 2 and GRK 6, which can lead to the phosphorylation of Mu opioid receptors. Moreover, GRKs and receptor phosphorylation leads to the recruitment of β-arrestin 2 causing desensitization of mu opioid receptors through internalization and degradation. Abbreviations: Mu, mu opioid receptor; G-proteins, guanine nucleotide-binding proteins; red and green arrows, increase and levels, respectively; GRK, G protein-coupled receptor kinase. (For interpretation of the references to colour in this figure legend, the reader is referred to the Web version of this article.)

Several studies from heroin users have revealed biochemical alterations in the striatum and prefrontal cortex (Table 2 and summarized in Fig. 3) of heroin abusers (Ferrer-Alcon et al., 2004; Sillivan et al., 2013). Biochemical analyses of postmortem brains from heroin users showed abnormal protein levels of mu opioid receptors in the putamen (Sillivan et al., 2013) and prefrontal cortex (Ferrer-Alcon et al., 2004). Tissue from prefrontal cortex of heroin abusers also showed increased protein expression of G protein-coupled receptor kinase (GRK) GRK2, GRK6 and β-arrestin-2 (Ferrer-Alcon et al., 2004). Several reports from postmortem cortical tissue found decreased protein expression of Protein Kinase C-α (PKCα) (Busquets et al., 1995; Garcia-Sevilla et al., 1997) and adenylyl cyclase-I (Shichinohe et al., 1998, 2001). Postmortem tissue from heroin users showed increased abundance of guanine nucleotide binding proteins subunits: Gαi_1/2_, Gαo, Gαs and Gαβ (Busquets et al., 1995; Escriba et al., 1994; Garcia-Sevilla et al., 1997; Hashimoto et al., 1996). These findings support the notion that heroin intoxication causes several neurobiological abnormalities in the prefrontal cortex (Ferrer-Alcon et al., 2004) and striatum (Sillivan et al., 2013).

**Table 2:** Postmortem brains of heroin addicts.

Region	Biochemical alterations	References
Prefrontal Cortex	Decrease PKC-α, increase Gαi1/2	Garcia-Sevilla et al. (1997)
Striatum	Decreased ERK and ELK-1	Sullivan et al. (2013)
Temporal Frontal Cortex	Decreased adenylyl cyclase	Shichinohe et al. (2001)
Nucleus Accumbens	Decrease Gαi1/2	McLeman et al., 2000
Prefrontal Cortex	GRK 2 (membrane associated)	Ozaita et al., 1998
Prefrontal Cortex	Decrease mu opioid receptors, increase GRK 2, increase GRK 6 and increase β-arrestin-2	Ferrer-Alcon et al. (2004)
Prefrontal Cortex	Increase in Gαi1/2, GαO, GαS, and Gαi	Escriba et al. (1994)
Prefrontal Cortex	Decrease PKCα, increase in Gαi1/2	Busquest et al. (1995)
Temporal Frontal Cortex	Decrease adenylyl cyclase-I	Shichinohe et al. (1998)
Temporal Frontal Cortex	Increases Gβ subunits	Hashimoto et al. (1996)
Orbital Frontal Cortex	Altered DNA methylation	Kozlenkov et al., 2017

### 4.3 Heroin effects on neurocircuits

Heroin dependency is associated with defects in the temporal, frontal, and anterior cingulate regions of the cortex, which has negative consequences to glutamatergic efferents, from the frontal cortex to the nucleus accumbens (Kalivas, 2004; W. Wang et al., 2010). Clinical evidence from heroin abusers found decreased functional connectivity between the frontal cortex and nucleus accumbens (W. Wang et al., 2010; Zou et al., 2015). Resting-state functional MRI from heroin-dependent patients showed defects in connectivity between the prefrontal cortex and nucleus accumbens (Zou et al., 2015). Furthermore, heroin addicts showed abnormal functional connectivity in the basal ganglia and amygdala (Ma et al., 2010; Upadhyay et al., 2010). In patients who had abstained from heroin were found to have abnormal connectivity between the nucleus accumbens and amygdala (Xie et al., 2014), suggesting this regions may be related to withdrawal symptoms. In the same study, neural connectivity defects were also observed between the thalamus and the prefrontal cortex, which neurocircuits have been implicated in the process of decision-making and habit learning/reward as depicted in Fig. S3.

The data reviewed here indicate that heroin intoxication is accompanied by biochemical changes in the abundance of mu opioid receptor. Although the underlying mechanisms of heroin addiction are still unknown, this review revealed a potential mechanism that involves signaling through mu opioid receptor and intracellular proteins downstream of this receptor (see Fig. 3 for summary). This statement is consistent with preclinical models of opioid self-administration (Blackwood et al., 2021). The involvement of mu opioid receptor in addiction is supported by reports that genetic mutations in OPRM1 increased vulnerability of heroin addiction in humans (Drakenberg et al., 2006) and in preclinical models (Sillivan et al., 2013). Moreover, the role of this receptor in addictive processes is supported by observations that an A118G single nucleotide polymorphism of mu opioid receptor is linked to opioid dependence (Bart et al., 2004) and alcoholism (Bart et al., 2005). In addition, a single nucleotide polymorphism found in the CREB gene was reported to correlate with heroin use (Pal et al., 2014), Also relevant to this discussion are findings that epigenetic factors that regulate the expression of mu opioid receptor are associated with heroin addiction. Specifically, there was increased methylation of cytosine residues in cytosine:guanine (CpG) dinucleotides clusters located in the OPRM1 promoter of former heroin addicts (Nielsen et al., 2009). Together, these observations suggest that mu opioid receptor-associated mechanisms may involve changes in signaling mechanisms downstream of CREB (Kumar et al., 2011; Lane-Ladd et al., 1997).

## 5 Methadone use disorder

In the 1960's, methadone was introduced as a treatment for opioid withdrawal symptoms (Dole and Nyswander, 1965). Subsequently, these methadone clinics were helpful in the treatment of opioid users infected with the human acquired immunodeficiency (HIV). Methadone is a synthetic opioid that is commonly administered orally as a racemic mixture of two enantiomers: R-methadone (R-Met) and S-methadone (S-Met) (Foster et al., 1999; Inturrisi, 2005). Methadone is one the most long-lasting opioid medication with a half-life of approximately 13–50 hours. The drug also has an affinity for the NMDA (N-methyl-D-aspartate) receptor, an inotropic glutamate receptor, which plays a critical role in modulating long-term and memory formation in addiction (Bauer et al., 2002). Methadol, the active metabolite of methadone, causes analgesia, sedation, and euphoria. The negative side effects include itching, nausea, and respiratory depression.

Methadone usage has increased, in large part, due to growing numbers of treatment programs that offer methadone maintenance therapy (MMT) (Kreek et al., 2019). MMT is one of the few effective treatments for people suffering from OUD (D'Aunno et al., 2014). In fact, several studies have reported that MMT correlated with a reduction in heroin-related deaths (Caplehorn et al., 1994; Gronbladh et al., 1990). Successful use of MMT requires trained medical staff because methadone remains a controlled substance. Furthermore, unsupervised usage of methadone has caused overdose deaths and serious neurological damage (Drummer et al., 1992; Hedegaard et al., 2020; Wu and Henry, 1990).

### 5.1 Biochemical and structural abnormalities

In children, acute methadone toxicity can cause brain abnormalities. T2-weighted images from magnetic resonance imaging (MRI) found lesions in the cerebellar hemispheres (Hosseini, 2017). This finding is consistent with reports of leukoencephalopathy (Anselmo et al., 2006; Metkees et al., 2015; Tiong et al., 2019) and cerebellar edema (Hosseini, 2017) in methadone users. Other defects in cerebellar hemispheres include hyperintensities in white matter (Metkees et al., 2015) and hydrocephalus (Anselmo et al., 2006; Metkees et al., 2015). Structural abnormalities were also found in the U-fibers of the cortex (Metkees et al., 2015). Moreover, brain MRI detected lesions in the hippocampus (Anselmo et al., 2006). In contrast to children, adults show very little severe neurological conditions. However, computed tomographic scans showed cerebellar edema in adult patients that inhaled methadone (Palmiere et al., 2011; Winklhofer et al., 2014).

In children and young adults, methadone exposure causes widespread defects in the cortex (Metkees et al., 2015; Tiong et al., 2019), subcortical U-fibers (Metkees et al., 2015), cerebellum (Hosseini, 2017; Metkees et al., 2015; Reisner et al., 2015), hippocampus (Anselmo et al., 2006), and other white matter regions (Metkees et al., 2015). Hence, special consideration should be taken to limit the exposure of methadone to children, young adults, and pregnant women. Methadone-induced lesions in the cerebrum and cerebellum were found to be reversible, suggesting that patients may recover over time (Cerase et al., 2011). The etiology of methadone-induced lesions remains unknown. However, these are thought to be secondary to hypoxia-induced leukoencephalopathy or atrophy. Long-term use of methadone has indeed been reported to cause damage to white matter in patients maintained on the drug for therapeutic purposes (Li et al., 2016). Additional studies examining the neurological consequences of long-term use of opioids for maintenance therapy may be necessary in order to develop recommendation for possible monitoring of these patients in medical settings.

## 6 Fentanyl use disorder

Fentanyl or its derivatives have been used by physicians for pain management and for treatment in opioid-tolerant patients (Schifano et al., 2019). However, in recent years the U.S. spike in opioid-related deaths are due to illicit use of fentanyl and fentanyl-related analogs (Blackwood and Cadet, 2021; Rudd et al., 2016). The effects of fentanyl include sedation, nausea, and respiratory depression. Fentanyl also causes neurotoxic psychological symptoms in the form of visual hallucinations, insomnia, and anxiety (Ostwal et al., 2015). Furthermore, these compounds have the potential to adversely impact the respiratory and immune systems (Bidlack et al., 2006; Tabatabai et al., 1989). Human studies have reported that high doses of fentanyl lead to respiratory depression (Mildh et al., 2001), likely from its effect on respiratory neurons located in the brainstem (Yuge et al., 1985)

Fentanyl and fentanyl analogs are potent mu opioid receptor agonists. For example, fentanyl is approximately 80–100 times more potent than morphine. A fentanyl-derived drug, carfentanil, has approximately 10,000 more affinity to mu opioid receptor than morphine (Meert et al., 1988; Van Bever, Niemegeers, Schellekens and Janssen, 1976). Fentanyl and fentanyl analogs undergo biotransformation by CYP enzymes into inactive metabolites (Feasel et al., 2016; Tateishi et al., 1996).

### 6.1 Biochemical and structural abnormalities

Acute fentanyl intoxication cause spongiform leukoencephalopathy in toddlers (Foy et al., 2011) and adult patients (Switzer et al., 2020). MRI and histological findings showed cerebral hemorrhage (Helander et al., 2017) and cerebral edema (Freni et al., 2019; Geile et al., 2019; Guerrieri et al., 2017; Schonfeld et al., 2019) in the majority of fentanyl and fentanyl-related clinical cases (Fomin et al., 2018). Fentanyl users also had hyperintensities in the hippocampus (Switzer et al., 2020). MRI in preterm infants found cerebellar injury and cerebellar hemorrhage that correlated with high dosages of fentanyl (McPherson et al., 2015).

## 7 Conclusions

In this review, we summarized the structural abnormalities reported after acute and chronic opioid exposure. The accumulated evidence supports the notion of potential opioid-induced neuropathological damage in discrete brain regions that serve as substrates for the cycle of opioid use disorder. The further identification of potentially vulnerable brain regions in these patients might serve as guides for optimal implantation sites for deep brain stimulation technology, one of the latest tools being explored to treat opioid addiction (Krauss et al., 2021) because abnormalities in these areas might regulate the vicious addiction process.

Finally, Fig. 3 represents a schematic rendering for the potential involvement of signaling factors downstream mu opioid receptors in various brain circuits implicated in substance use disorders. Activation or inhibition of these signaling cascades might lead to drug-induced epigenetic alterations that include DNA methylation and histone post-translational modifications. Indeed, DNA samples taken from patients suffering from heroin use disorder were found to have increased levels of DNA methylation (Nielsen et al., 2008; Xu et al., 2018). Aberrant alterations in DNA methylation are associated with various neuropsychiatric diseases, including heroin use disorder (Cadet and Jayanthi, 2021; Nielsen et al., 2008; Wang et al., 2016).

## Data availability statement

Data sharing not applicable to this article as no datasets were generated or analyzed during the current study.

The authors declare no other relevant competing interests that could have appeared to influence the work reported in this review.

## Funding

Work in Dr. Jean Lud Cadet's laboratory is supported by funds of the Intramural Research Program of the 10.13039/100000016DHHS/10.13039/100000002NIH/10.13039/100000026NIDA.

## CRediT authorship contribution statement

**Christopher A. Blackwood:** Data curation, Visualization, Writing –original and revised manuscript. **Jean Lud Cadet:** Conceptualization, preformed writing –review and editing.

## Declaration of competing interest

The authors declare that they have no known competing financial interests or personal relationships that could have appeared to influence the work reported in this paper.
