# Supplementary material for: The molecular neurobiology and neuropathology of opioid use disorder
Source: Curr Res Neurobiol. 2021 Oct 14;2:100023. doi: 10.1016/j.crneur.2021.100023 (PMC9090195; doi:10.1016/j.crneur.2021.100023)
Supplement: Multimedia component 1 [file mmc1.docx]

**Supplementary Information**

**The Molecular Neurobiology and Neuropathology of Opioid Use Disorder**

Christopher A. Blackwood and Jean Lud Cadet

|  |  |
| --- | --- |
|  | **Table S1. DSM-V Criteria for Opioid Use Disorder (OUD)*** |
|  | Consumption of large quantities of opioids |
|  | Strong desire or failure in an effort to cut down or control opioid use |
|  | Excessive time spent getting, using, or recovering from its effects. |
|  | Strong desire or cravings to use an opioid |
|  | Opioid use negatively impacts duties at work, school, or home |
|  | Continued opioid use despite having constant social or interpersonal problems exacerbated by the effects of opioids |
|  | Lost or reduced social, occupational, or recreational activities because of opioid use |
|  | Opioid use in situations in which it is physically hazardous (e.g. operating a car) |
|  | Continued opioid use despite recurrent physical or psychological problem (e.g. legal problems) |
|  | Need for increased doses of an opioid for effects, diminished effect per dose or both^#^ |
|  | Consumption of drugs to avoid withdrawal symptoms or withdrawal when dose of opioid is reduced^#^ |
|  | *Mild OUD, if two or three items occur in the same year. Moderate OUD, if four or five items occur. Severe OUD, if six or more items occur. ^#^If the opioid is taken only as prescribed, this item does not count towards a diagnosis of an OUD. Criteria are from the *Diagnostic and Statistical Manual of Mental Disorder*, fifth edition (DSM-V, 2013) |

|  |
| --- |

**
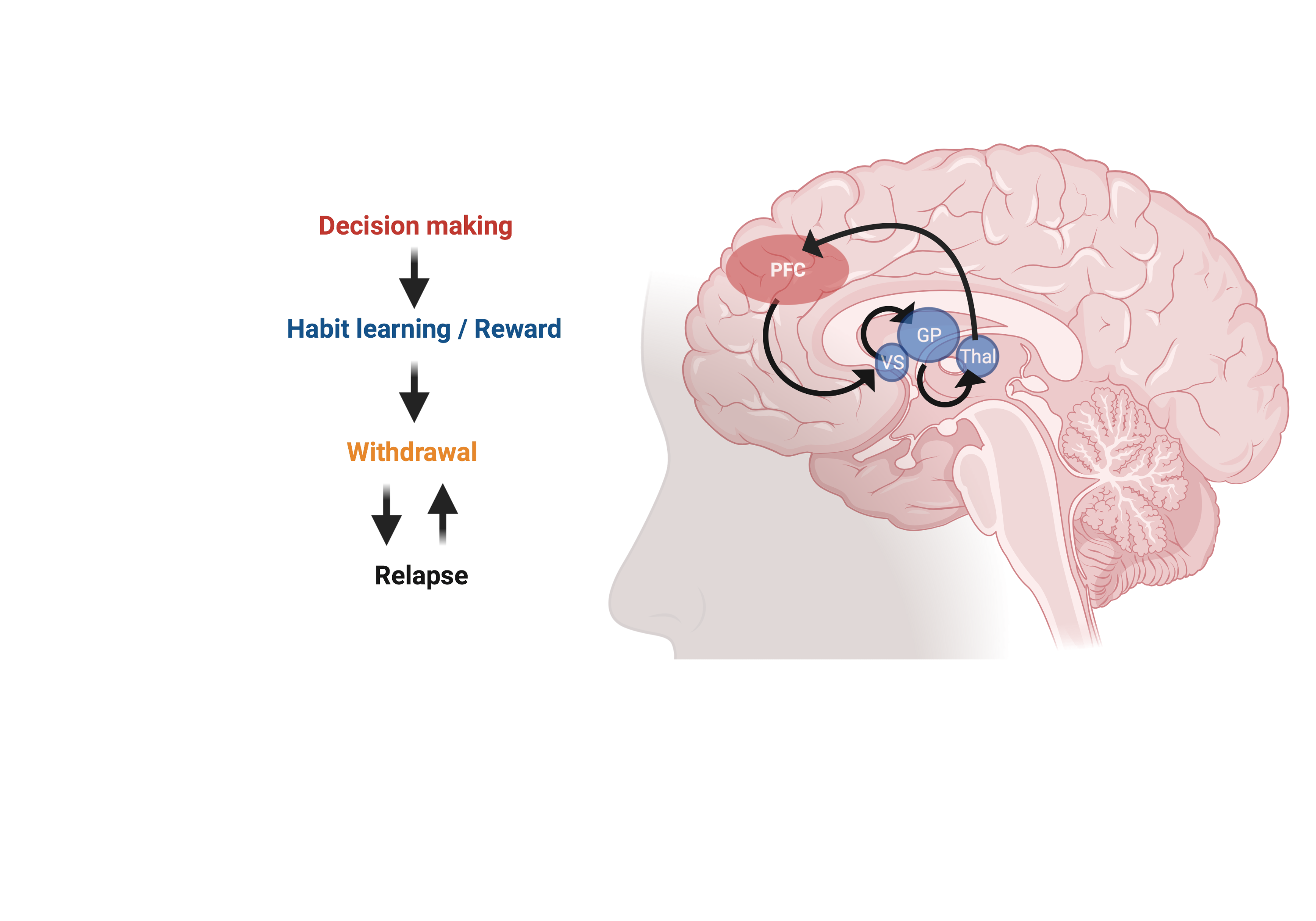
**

**Figure S1. Potential oxycodone disrupted neurocircuitry.** Sagittal view of a cartoon human brain showing regions of the brain disrupted by oxycodone (red and blue circles) and neurocircuits (black arrows) associated with decision making and habit learning/reward involved in the progression to chronic relapses. Abbreviations: GP, Globus Pallidus; PFC, Prefrontal Cortex; Thal, Thalamus; VS, Ventral Striatum.

**
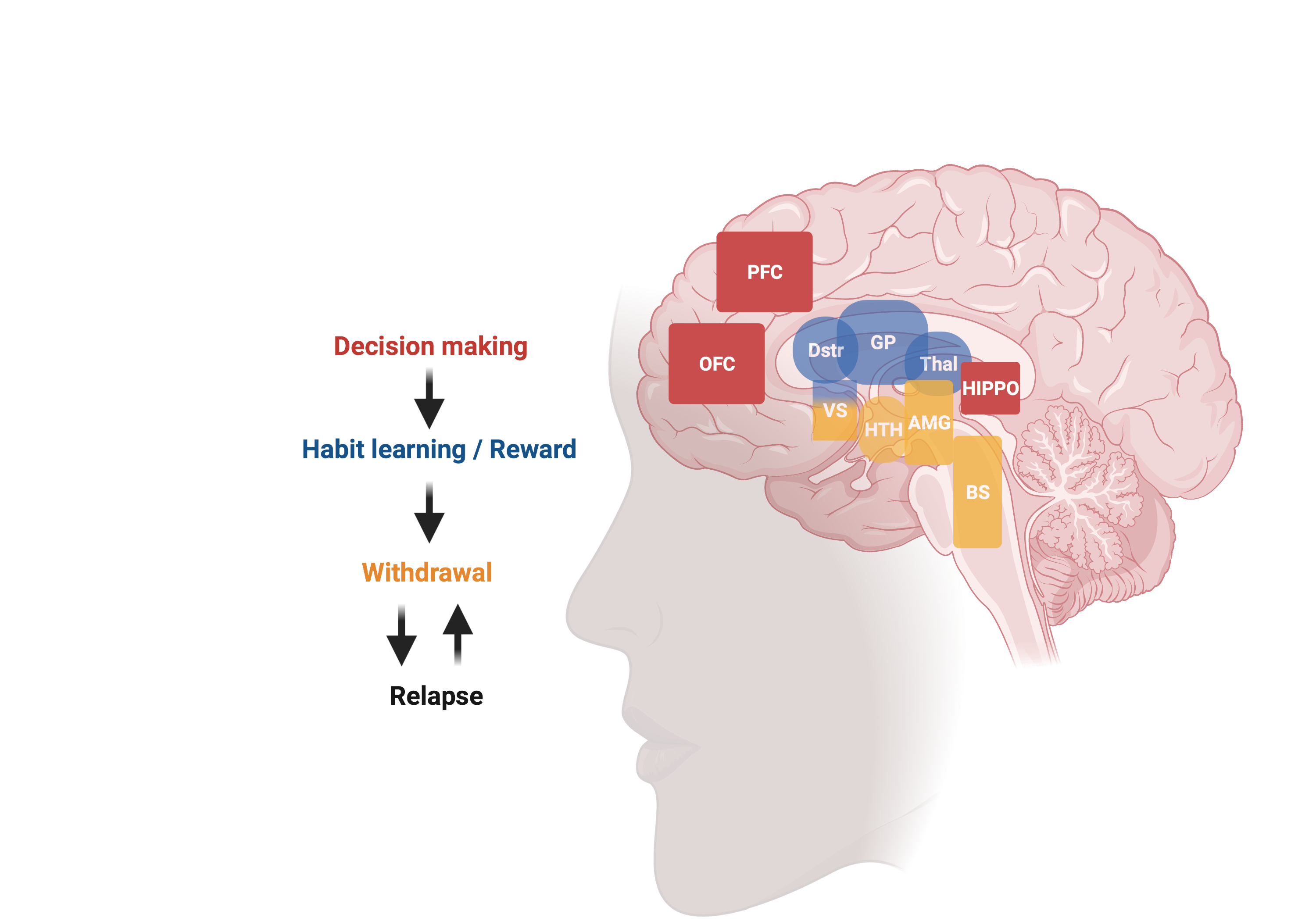
**

**Figure S2. Brain regions linked to the progression of chronic relapse.** Diagrams of the brain regions associated with the progression to chronic relapses. Decision making process involves the initial exposure to opioids. The regions associated with decision-making stage involve the hippocampus, orbital frontal cortex, and prefrontal cortex (red boxes). Habit learning/reward stage is linked to the increasing of opioid doses and the escalation of drug taking and includes globus pallidus, dorsal striatum, ventral striatum, and thalamus (blue circles). Withdrawal stage involves the hypothalamus, brainstem, ventral striatum, and amygdala (orange rectangles). Abbreviations: PFC, Prefrontal Cortex; OFC, Orbital Frontal Cortex; Dstr, Dorsal Striatum, GP, Globus Pallidus; Thal, Thalamus; AMG, Amygdala HTH, Hypothalamus; VS, Ventral Striatum; Hippo, Hippocampus; BS, Brain stem.

**
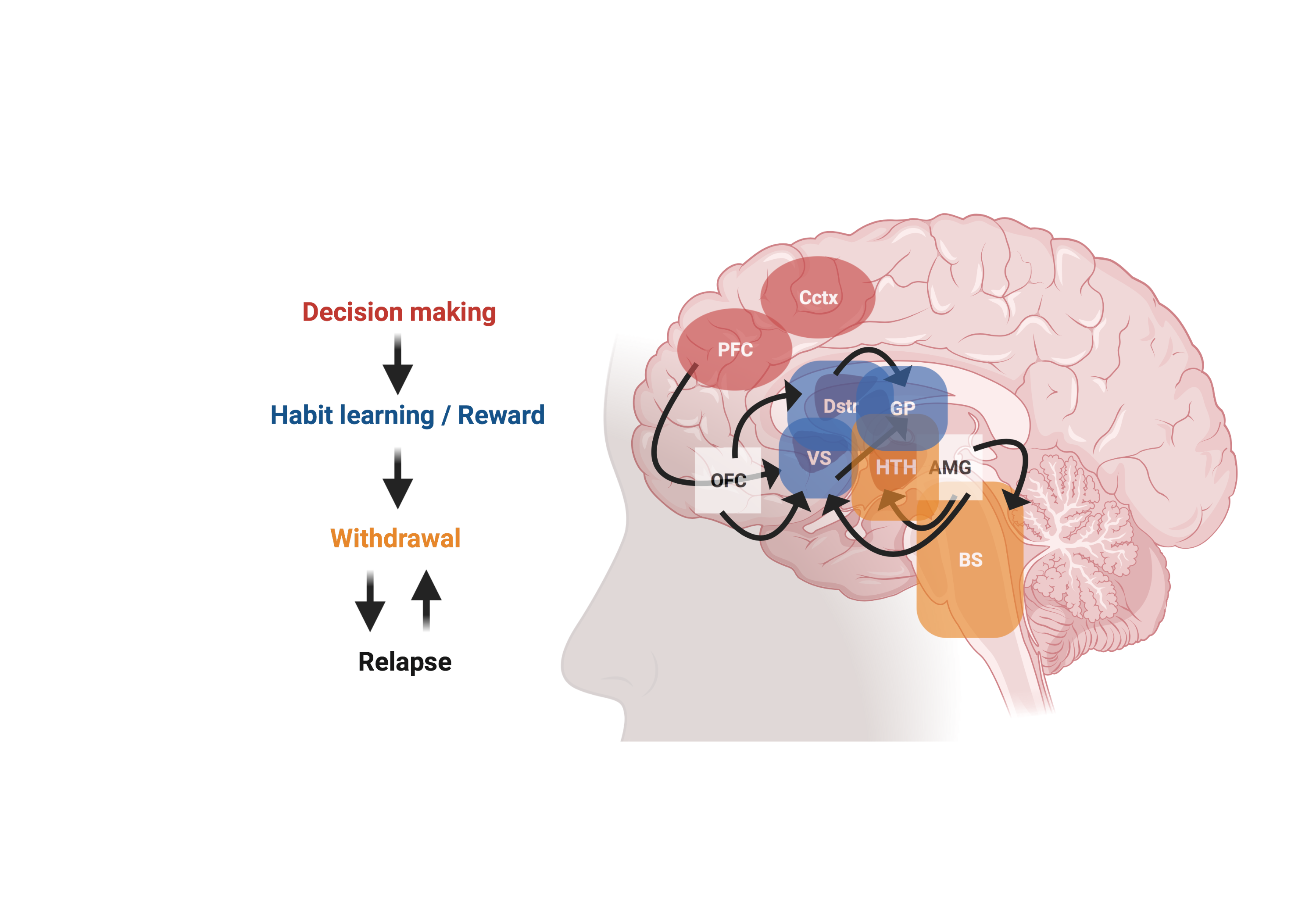
**

**Figure S3. Potential heroin disrupted neurocircuitry.** Diagram of the human brain showing regions (red circles, blue boxes, orange rectangles) of the brain disrupted by heroin and neurocircuits (black arrows) associated with these regions. Abbreviations: OFC, Orbital Frontal Cortex; PFC, Prefrontal Cortex; Cctx, Cingulate Cortex; Dstr, Dorsal Striatum; GP, Globus Pallidus; AMG, Amygdala; HTH, Hypothalamus; VS, Ventral Striatum; BS, Brain stem

**References**

DSM-V. (2013). Diagnostic and Statistical Manual of Mental Disorders, American Psychiatric Association. (Fifth Edition ed.).
